# Motivation for and Challenges in Teacher Research in Underdeveloped Areas of Northwest China: An Exploratory Study

**DOI:** 10.3390/bs14111064

**Published:** 2024-11-07

**Authors:** Na Zhou, Xin Liu, Xinglin Jin, Tongji Li, Chenjing Wang, Wilfried Admiraal

**Affiliations:** 1 Institute of Vocational and Technical Education (CDIBB), Tongji University, Shanghai 201804, China; zhoun@tongji.edu.cn (N.Z.); litj@tongji.edu.cn (T.L.); 2 Department of Teacher Education and School Research, Faculty of Educational Sciences, University of Oslo, 0317 Oslo, Norway; 3 School of Education Science, Jiangsu Normal University, Xuzhou 221116, China; jinxl1990@163.com; 4 Department of Education, Xiaodao Educational Development Institution, Xining 810007, China; 13897580551@163.com; 5 Centre for the Study of Professions, Oslo Metropolitan University, 0167 Oslo, Norway; wilfried@oslomet.no

**Keywords:** VTs, LPA, self-determination

## Abstract

This study explored the motivations and challenges vocational teachers (VTs) face in conducting research in underdeveloped regions of Northwest China. We invited 49 vocational teachers from Qinghai province to participate in the questionnaire survey, with their motivation measured using scale items and their challenges measured using open-ended questions. After data collection, latent profile analysis (LPA) was used to explore the participants’ motivational profiles, and three types were identified, i.e., high autonomous and controlled motivation, high autonomous and low controlled motivation, and low autonomous motivation. In addition, we conducted a qualitative analysis of the challenges in teacher research. As a result, five categories of challenges that might hinder Chinese vocational teachers in conducting research were observed (i.e., researcher identity, research knowledge and skills, research climate in schools, workload and family care, and resources and financial support). Teachers with the profile of highly autonomous and controlled motivation were more likely to face challenges related to a lack of resources and financial support and the research climate. Workload and family care appeared to be significant challenges for teachers with the profile of highly autonomous but less controlled motivation. In contrast, a lack of research knowledge and skills was a common perceived challenge across all profiles. These results suggest that although vocational teachers express relatively high motivation in conducting research, the significance of institutional development programmes and external research support for research activities remains crucial.

## 1. Introduction

Most countries view research activities as a voluntary endeavour teachers conduct for professional growth or academic recognition [1]. In China, working on research used to be a central component of faculty responsibilities at universities, often linked to career advancement and institutional reputation. However, the expectation to engage in research has gradually expanded beyond universities and into other types of education. Vocational education (in China, vocational education is structured into two distinct systems: secondary vocational education and higher vocational education. Secondary vocational education typically begins after students complete compulsory education (usually after lower secondary school) and focuses on a blend of academic instruction and practical skill development. The aim is to prepare students for their entry into the workforce or further education [2]. Higher vocational education is an integral part of the higher education system in China, similar to community colleges or technical institutes in other countries [3]. These institutions provide post-secondary education, offering advanced technical and professional training aiming to prepare students for specialised roles in the labour market [4]. Higher vocational institutions confer associate or bachelor degrees, positioning themselves as crucial players in China’s economic and workforce development [5] in particular has seen a significant increase in research demands, with teachers now required to integrate research activities alongside their teaching duties. This trend is evident among teachers at both secondary and higher vocational education levels (both secondary vocational teachers and higher vocational teachers are collectively referred to as vocational teachers in China. While their teaching contexts differ, with secondary vocational teachers focusing more on foundational skills and higher vocational teachers on more advanced training, they are united by their roles in vocational education and increasingly by the expectation for them to engage in research activities [6]. Throughout this paper, the term “vocational teachers” refers to educators from both of these systems unless otherwise specified), where there is a growing expectation of them to contribute research in addition to performing their teaching roles [3,7,8,9].

Traditionally, Chinese vocational education focused on practical skill training to meet labour market needs [2,4,5]. In recent years, reform within education has called for embedding research into vocational education and making it an integral part of general efforts to upgrade the quality of vocational education to meet the country’s economic and innovative development demands [4,5]. For vocational teachers, particularly those transitioning from industry without formal training on educational research, these increased research expectations present distinct challenges. In less developed regions, these challenges are compounded by limited institutional support, scarce funding, and minimal access to mentorship, often rendering research engagement a seemingly unattainable task [10].

Despite challenges and some resistance to these demanding research requirements, there are notable benefits to embedding research within vocational education. Since teacher research is inherently focused on teaching practice, engaging in research could offer vocational teachers opportunities to enhance their awareness of educational methods and improve their teaching skills and quality. Specifically, conducting research on the issues they frequently encounter may inspire vocational teachers to develop effective solutions, making their practice more reflective and adaptive.

Furthermore, cultivating a research culture in vocational colleges aligns with China’s broader innovation-driven development strategy, positioning vocational education as central to addressing the nation’s economic and technological needs [11]. However, despite these benefits, little is known about the specific motivations and challenges vocational teachers face in undertaking research. To address this gap, this study explores the motivations driving vocational teachers at both secondary and higher levels to engage in research, while examining the obstacles they encounter and the possible relationships between their motivations and challenges.

Self-determination theory (SDT) offers a valuable framework for analysing vocational teachers’ research motivations. SDT helps distinguish between autonomous and controlled motivation, offering insights into how these factors influence teachers’ engagement with research. While existing studies have largely focused on general education teachers in developed regions, vocational teachers in underdeveloped areas remain understudied. Applying SDT in this context addresses this gap, providing theoretical insights and practical recommendations to support vocational teachers’ research engagement.

The initial findings could provide us with a potential explanation and a call for future research to sustain vocational teachers’ engagement in conducting research and inspire us to consider how to support vocational teachers’ professional growth and help them overcome the challenges posed by these dual responsibilities, thereby contributing to both their career development and the advancement of vocational education.

## 2. Motivation and Challenges for Teacher Research

In the past forty years, teacher research has been spread in the field of education. At present, it has been commonly involved in both pre-service teacher education programmes and in-service teacher professional development initiatives. Prior studies have demonstrated the positive effects of teacher research, including changing teachers’ cognition and attitudes [8,12,13], enhancing their efficacy [14], and fostering critical reflection in classroom practice [15,16,17]. Additionally, teacher research has been associated with broader improvements in school development and student outcomes [18,19,20]. Given these advantages, the global conversation among policymakers, school leaders, and researchers has increasingly focused on promoting teachers’ engagement in research. To address this issue, it is essential to use a person-centred approach [21] to consider the motivations that drive teachers to engage in research and the challenges they face that hinder their engagement in research activities at work.

### 2.1. Motivation for Teacher Research

Motivation is defined as the “reasons that underlie behaviour that is characterized by willingness and volition” [22]. It has been proven to be a crucial factor that can influence individuals’ attitudes, behaviours, and performance (e.g., [23,24]). Regarding teacher research motivation, Borg (2006) noted in his study that, “Teachers must have a reason for wanting to engage in teacher research” [25]. In other words, teachers will not conduct research unless they are motivated [26]. A growing body of work has found that teachers’ motivation for conducting research has an important influence on their research involvement and performance (e.g., [27]). Based on previous studies from different countries, teachers’ motivations to conduct research were reflected in how they perceived its value in terms of improving their teaching practice, their interest in research, and their professional development needs [28,29]. In contrast with Western countries, vocational teachers in Asian countries are experiencing higher policy pressures, and this is particularly the case in China. As a result, the motivations driving Chinese teachers to engage in research remain unclear. As Zhang (2021) indicated, whether teachers conducted research voluntarily or compulsively is not known [30]. Chinese vocational teachers are facing pressure from policymakers and school leaders. However, it is unclear whether these are the primary motivating factors (i.e., extrinsic motives) for vocational teachers to conduct research in their careers.

Therefore, to deconstruct Chinese vocational teachers’ motivation for research, we draw on self-determination theory (SDT), which refers to “an approach to human motivation and personality that uses traditional empirical methods while employing an organismic metatheory that highlights the importance of humans’ evolved inner resources for personality development and behavioural self-regulation” [31]. Before now, SDT, a dimensional theoretical framework that emphasises both the quantity and quality of individual motivation for work [32], has been broadly utilised to explore teachers’ motivation in teaching and professional learning [33,34] in Western cultures. It distinguishes five types of motivation, varying according to the degree of an individual’s self-regulation, i.e., intrinsic motivation, external regulation, introjected regulation, identified regulation, and integrated regulation [35]. External regulation and introjected regulation are often conceptualised as controlled motivation, which means that individuals’ actions are controlled by external pressure, while identified regulation and intrinsic motivation are usually combined to reflect autonomous motivation, which concerns individuals’ volitions and interests [36,37,38]. Based on SDT, the previous findings on teachers’ motivation for research can be understood systematically using self-reported and culturally validated psychometric surveys. For example, a study found that Singaporean teachers thought that carrying out research had limited utility for their teaching but that they were expected to be involved in research by their school leaders [28]. This can be interpreted as the teacher participants having a lower perception of autonomous motivation but a higher perception of external regulation.

### 2.2. Challenges in Teacher Research

Challenges to Chinese vocational teachers’ research engagement may be highly contextual and situational, but this is underdiscovered. Prior research from Western countries has explored challenges to teachers engaging in research, such as teachers’ professional status [39], ethical awareness [40], and time constraints [41]. Regarding China, although many studies have indicated that Chinese vocational teachers have difficulties in conducting research, few of them have revealed specific difficulties from an empirical perspective. Thus, to explore Chinese teachers’ challenges in conducting research, we should go deeper into the context of teacher research in China, which is still an emerging activity.

Chinese vocational teachers, particularly those in underdeveloped regions, face even more substantial challenges. There are over 490,000 primary and secondary schools as well as colleges and universities across 34 provincial regions of China, with significant disparities in their resources and infrastructure. In contrast with developed areas, such as Zhejiang and Jiangsu, teachers in underdeveloped areas, such as Qinghai and Gansu, experience greater difficulties due to poor economic conditions and weaker educational systems, which limit their capacity to engage in research. Vocational teachers in particular face unique challenges. This is because firstly, many vocational teachers come from industry backgrounds and lack formal teaching training, limiting their understanding of theoretical knowledge and skills about teaching and learning to support them in research. Secondly, there are more vocational teachers without a bachelor’s degree or higher than general teachers. As recent statistics from the Ministry of Education showed, the percentage of vocational teachers was around 7%, while for general teachers, it was only around 1% in secondary education [42]. Thirdly, the population within vocational education is different from that of general education, with more students with behavioural problems and special needs, which makes the additional effort of conducting research more complicated [43,44]. However, conducting research is particularly important for vocational teachers. As mentioned above, compared to general education, students in vocational education often have worse academic performance and self-control. This means vocational teachers have much more difficulties in working on classroom management and teaching practice. Based on teacher research, vocational teachers could obtain a more in-depth understanding of their students, especially problematic students, and may find more effective approaches to improving their teaching performance or helping students to make progress.

Based on the above, in our study, we would like to contribute insights into teachers’ motivation for and challenges in engaging in research within the context of vocational education within underdeveloped areas of northwest China. By focusing on this group, this study aims to provide an exploratory understanding of teacher research and provide practical insights for policymakers and school leaders seeking to support vocational teachers in their dual roles as teachers and researchers. The following three research questions were pursued in this study:

Q1: What motivates Chinese vocational teachers from underdeveloped areas to participate in educational research?

Q2: What challenges impede Chinese vocational teachers from engaging in educational research?

Q3: How are vocational teachers’ motivations and challenges related regarding conducting research?

## 3. Method

### 3.1. Participants

The participants were 49 Chinese vocational teachers from Qinghai province. There were 13 males and 36 females, aged from 23 to 51. The average teaching experience of the participants in years was 11.37, and 13 vocational teachers had gained teaching experience for less than five years. The sample reflected various teaching subjects (e.g., computer science, electronic commerce, and nursing).

### 3.2. Procedures

The data were collected from the programme “Project for Improving Vocational Teacher Quality”, which was funded by the Qinghai government. There were 49 vocational teachers from different cities in Qinghai province. At the start of this project, the first author requested that the participants fill out a questionnaire containing pre-structured items related to research motivation. It was explained to the participants that their participation was voluntary and that the data would be kept confidential and only made available for research purposes. Also, it was explained that the survey would not influence the assessment of their learning in this programme. By completing the questionnaire, the participants gave their consent to participate. After the participants had finished the survey, they were asked to fill out another questionnaire containing open-ended items related to their latest research experience. Of these 49 participants, 43 completed the first questionnaire measuring motivation for research at work, and 35 completed the second questionnaire related to their research challenges (29 completed both).

### 3.3. Measures

#### 3.3.1. Motivation for Conducting Research

The teachers’ motivation for undertaking educational research was measured in the first questionnaire using a scale adapted from Vansteenkiste et al. (2009), which focused on students’ motivation to learn [45]. There were four constructs, i.e., intrinsic motivation (engaging in education research for pleasure), identified regulation (engaging in education research when they fully perceived its importance), introjected regulation (engaging in education research due to internal or esteem-based pressures), and external regulation (engaging in education research to obtain external incentives or avoid punishment). Each construct consisted of 4 items, and the teachers rated their agreement with each item using a scale ranging from 1 (“Totally disagree”) to 5 (“Totally agree”). Since the scale from Vansteenkiste et al. (2009) was not used to explore the teachers’ motivation for undertaking research [45], we conducted an exploratory factor analysis (EFA) with varimax rotation to test its construct validity. The results showed that twelve items (at three items for each construct) were well loaded onto their expected original constructs, and four items were deleted because of cross-loading. The final scale explained a total of 81.20% of the variance. The Cronbach’s alphas for these four subscales were, respectively, 0.92, 0.81, 0.80, and 0.92. The six items of introjected regulation and external regulation were summed up for controlled motivation (Cronbach’s alpha = 0.73); at the same time, the six items of intrinsic motivation and identified regulation were summed up for autonomous motivation (Cronbach’s alpha = 0.87).

#### 3.3.2. Challenges in Conducting Research

To contextualise the participants’ challenges in conducting research, we first asked them to reflect on their latest research experience in the second questionnaire. Then, they were requested to write down what problems they encountered during this experience and how they solved those problems. Furthermore, they were requested to think about what hindered them from undertaking research. Teacher participants who did not have any research experience were only required to respond to the last question.

### 3.4. Data Analysis

To respond to the first question, we explored the data from the first questionnaire. First, we used descriptive statistics to provide insights into the vocational teachers’ motivation for engaging in research from the perspective of the constructs. Then, latent profile analysis (LPA) was utilised to identify potential profiles of the participants with regard to their motivation for educational research. Considering the more constructs were included, the more complex the comparisons would be; to reflect the main differences among the profiles better, we used two combined motivations (autonomous and controlled) instead of four specific types of motivation to conduct the LPA. There were several model fit indicators that were applied to choosing the most appropriate model with regard to the profiles. Specifically, entropy was used to measure the uncertainty of the classification, and values above 0.8 were considered good. Akaike’s information criterion (AIC), the Bayesian information criterion (BIC), and the sample-adjusted BIC (aBIC) were applied to compare the models, and lower values meant that the current model was closer to the true model than the last one. In addition, we employed the Lo–Mendell–Rubin Adjusted LRT (aLMR) and the bootstrap likelihood ratio test (BLRT) to test the improvement in the fit between neighbouring models. A significant P-value may suggest a possible improvement in the model fit compared to that of the previous model [46]. Although the sample was quite small, the results of the data analysis were quite convincing for the following reasons: First, the sample was distributed across various cities and schools, with different ages, levels of teaching experience, and teaching subjects, and could represent a real group of vocational teachers from Qinghai province. Second, as the number of motivational items included in our study was limited, there was a lower demand in terms of the number of participants. Third, since we considered this study an exploratory instead of a confirmatory study, a small group of participants was acceptable.

Table 1 displays the model fit statistics from one to four profiles. *Based on these indicators, the three-profile model appears to provide a reasonable representation of the data.*

To answer the second research question, the texts written by the teacher participants were transcribed ad verbatim and then explored to uncover common challenges. Fragments related to teachers’ challenges in conducting educational research were selected first. A fragment usually represented one coherent view, no matter its length. One fragment was selected per participant. Then, the fragments selected were coded using open coding. The categories were labelled based on the main challenges the teachers formulated. Third, the categories were merged and revised based on axial coding, leading to the construction of five categories (see Table 2).

In addressing the third research question, we analysed the responses from the 29 participants who provided insights related to both the motivations and challenges they encountered. Firstly, we divided these participants into the three motivational profiles we identified earlier. Then, their responses were categorised according to the challenges they faced in conducting research. Specifically, the five categories of challenges we summarised were used as a coding scheme and coded as 0 or 1, depending on whether the participants described them. Finally, we examined the frequency of each challenge category within the three profiles, which could suggest patterns in how the challenges aligned with different motivational profiles (shown in Table 3).

## 4. Results

**Figure 1:**
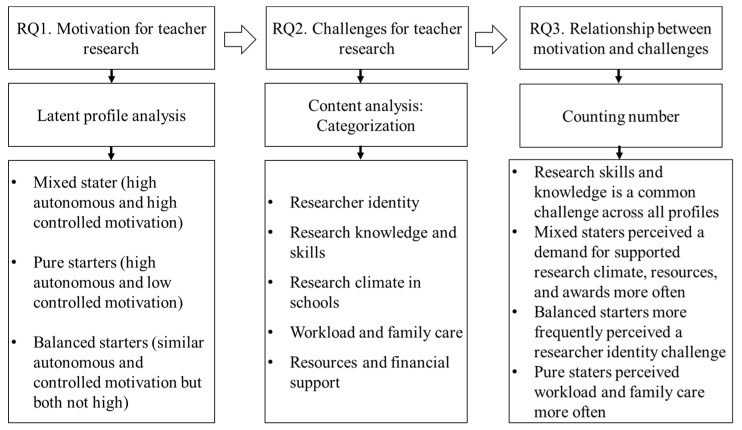
The presentation of the main results of this study.

As shown in Figure 1, we explored vocational teachers’ motivations and challenges in conducting research, as well as the relationship between these two factors. In general, three types of motivational profiles and five categories of challenges were identified. Also, several features of the relationship were summarised.

### 4.1. Motivation to Conduct Research

As shown in Table 4, the teacher participants scored highest on the construct of identified regulation (M = 4.36), followed by internal motivation (3.44), while they scored lowest on the construct of introjected regulation (M = 2.67), followed by external regulation (M = 2.97). In other words, the teachers’ autonomous motivation (a combination of internal motivation and identified regulation) was much higher than their controlled motivation in conducting research (a combination of external regulation and introjected regulation).

**Figure 2:**
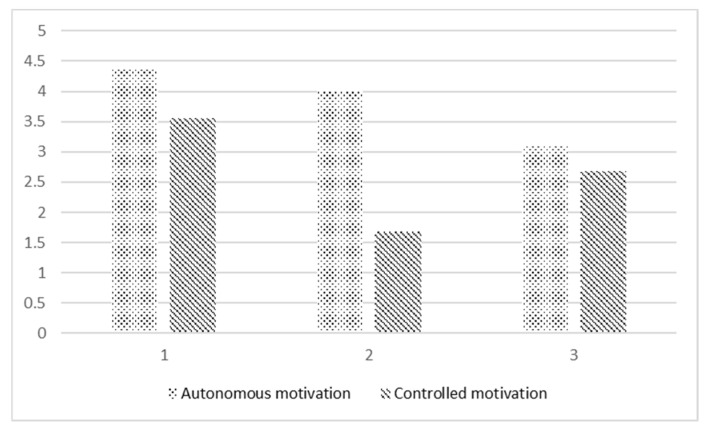
Means for the autonomous and controlled motivation in the three profiles.

Furthermore, the LPA generated three profiles of teachers regarding their perceived autonomous and controlled motivation. As shown in Figure 2, the teachers in the first profile showed high (i.e., way above the mean) autonomous and controlled motivation. There were 19 participants in this profile, and we labelled it as mixed starters. The second profile shared a similar level of autonomous motivation to that in the first one, while the mean score for controlled motivation was relatively low. The number of participants in this profile was 10, and we labelled this profile as pure starters. The third profile, with 14 participants, had much lower autonomous motivation among the three groups but a medium mean score for controlled motivation. Finally, we labelled the third profile as balanced starters. Interestingly, for all three profiles, the mean score for the teachers’ autonomous motivation was higher than that for their controlled motivation, with the largest differences in profile 2.

### 4.2. Perceived Challenges in Conducting Research

*Based on an exploratory approach to the analysis*, five categories of challenges to vocational teachers working on research were generated: (1) researcher identity; (2) research knowledge and skills; (3) research climate in schools; (4) workload and family care; and (5) resources and financial support.

#### 4.2.1. Researcher Identity

Six participants reported that their weak identity in terms of being a researcher was one of the biggest challenges that impeded them from working on educational research. Based on the explanation of the participants, we found that vocational teachers’ researcher identity was particularly influenced by their teacher identity for both novice teachers and experienced teachers. Specifically, for novice teachers, constructing teacher identity is often perceived as more important and pressing than developing their researcher identity. As a novice teacher participant said,

“I felt very confused for the past several months. I’m not sure if I am suited to be a teacher. Let alone doing research”.

It seems that novice teachers can only consider their identity as a researcher after they have adapted to their new role as a teacher.

Experienced vocational teachers seemed to perceive their teacher identity and teaching competency as more developed compared to novice teachers. Yet it might be that experienced teachers find it more comfortable to stay in their current situation and are afraid of extending their expertise. For example, an experienced teacher participant noted.

“I am very satisfied with my teacher role, and this to some extent inhibits me from trying a researcher role”.

#### 4.2.2. Research Knowledge and Skills

Research ability was the most frequent challenge, which was identified by 28 participants. Among these participants, nine of them stated that a lack of experience in conducting research prevented them from carrying out research, as they did not have any idea how to start. This lack of research knowledge and skills was mainly reflected in four aspects.

*Topic selection*. At the start of the research process, how to determine a topic was an important step for the vocational teachers. Nine participants mentioned that the selection of a topic was rather difficult for them. As a participant described,

“When I am seeking a research topic, I feel that there are so many topics that I can choose. However, when I focus on a specific topic, I do not know what to do with it. So, every time, I hesitate for a long time in topic selection”.

Although vocational teachers were usually clear about what was happening in vocational education based on their teaching experience, some of them were not able to translate those problems from their work reality into professional or academic research questions. This meant that they could not assess whether the research questions that they thought of were feasible to conduct research on either. The participants mentioned three concerns. First, they were afraid that a topic might be too general to conduct research on. Second, they were not sure whether a topic was innovative enough. Third, they did not know whether a topic had scientific significance.

*Theoretical knowledge.* In addition to topic selection, how to build a theoretical framework was also considered a difficult step for the vocational teacher participants. Five participants mentioned that substantial knowledge of educational concepts and theories is crucial for carrying out educational research. However, they also indicated that they had limited knowledge in this regard and they did not know how to search for them. Moreover, they also did not have any idea which concepts or frameworks were relevant to their research questions.

*Research methods.* Six participants stated that they had two types of worries about the research methods. The first one was that they could not find an appropriate method for answering their research questions, and the other one was that even if they chose a method, they did not know how to use it properly. A participant complained that this is like “wading across the river by feeling for the stones”. Some participants attributed this challenge to a lack of professional training on research.

*Report and writing.* Three participants mentioned that their writing was not academic, as they were not familiar with how to write in a structured way. They often copied sentences from journal articles and tried to adapt them to their contexts. For example, a participant described her writing experience as follows:

“Last time I only have half a month before the deadline for the submission of my project report. I did not know how to write it, only cut and paste from here and there.”

#### 4.2.3. The Research Climate in Schools

Twelve participants indicated that a weak research climate in school was the main problem with working on research. In their view, a good research climate is indicated by regulations and rules for undertaking research and high engagement in teamwork by vocational teacher colleagues. Unfortunately, most of the participants mentioned that they were not in such a climate, which was attributed to the underdeveloped economy in the region. This was explained by a participant:

“As our school is located in a remote district, the change is always delayed. It is hard to perceive the changes of new science and technology, and it makes us not have the spirit of exploration.”

Another participant mentioned that he had the feeling that high-quality Chinese educational journals were not willing to publish their research work because they were from vocational schools instead of universities.

#### 4.2.4. Workload and Family Care

Eleven participants reported that available time was a huge challenge to carrying out educational research, including teaching workload, non-teaching workload, and family care.

*Teaching workload.* Ten vocational teachers indicated that they were suffering with heavy teaching workloads, which meant that they did not have time and energy to conduct educational research. Differently from general teachers, they usually teach multiple subjects in a semester, which means they spend a lot of time teaching and preparing lessons. An interviewee who taught 18 lessons a week reported that together with preparing lessons and correcting students’ homework, there was little time to think about how to carry out research.

*Non-teaching workload.* In addition to teaching work, a substantial amount of non-teaching work took up a lot of the vocational teachers’ time and energy teachers in school, which was mentioned by five participants. For example, a headteacher in a class spent a lot of time on classroom management, communicating with parents, and preparing for inspections by school leaders or educational departments. Moreover, teachers are forced to undertake additional tasks due to the “double reduction” policy, which aims to relieve students’ burdens concerning homework and after-school tutoring. For example, one participant noted,

“Since the government published ‘double reduction’ policy, we are required to provide extra lessons for homework support or interest cultivation. Because of this, I always get home from work at seven or eight o’clock on workdays, and I feel so exhausted.”

*Family care*. This type of load was addressed by a female vocational teacher. As she explained, female vocational teachers usually have a stronger bond with their families, and they experience a higher family care load than male teachers. She concluded that this makes it harder to focus on educational research.

#### 4.2.5. Resources and Financial Support

The final category of challenges refers to a lack of resources and financial support for undertaking research, with two types of support: guidance from researchers and resources and rewards from the school.

*Guidance from academic researchers*. Six vocational teacher participants indicated that they could not receive effective guidance, and they believed carrying out research would be easier if they received more guidance. One participant reported that his school had no connections with universities, which made it difficult to receive guidance from academics.

*Resources and rewards from schools.* Several teacher participants mentioned that there was no equipment or resources to support them in conducting research and no mechanism for rewarding the completion of research in vocational schools. And they were reportedly not able to obtain enough financial support.

### 4.3. Mapping the Teachers’ Motivations and Challenges in Carrying Out Research

**Figure 3:**
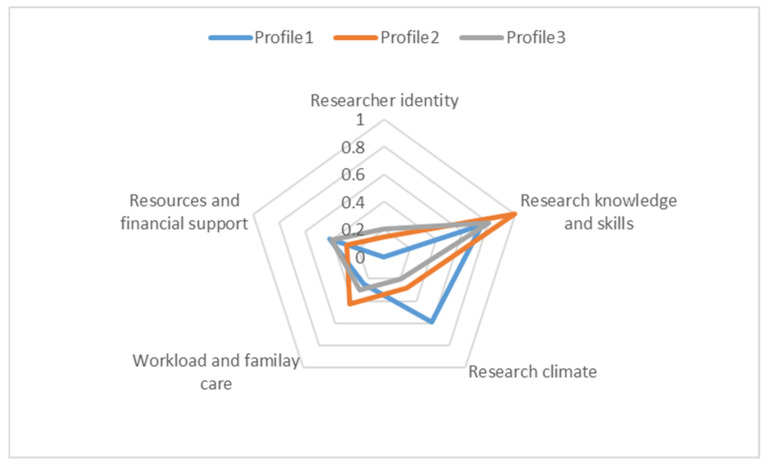
The frequencies of the challenges in the three profiles.

In addition to identifying the vocational teacher participants’ motivations and challenges in undertaking research, we also examined the possible relationship between the two. The average frequencies of the five challenge categories arising among three motivational profiles are displayed in Figure 3. Based on this, several findings were captured. First, the challenge of developing a researcher identity was most frequently reported by the participants from the third motivational profile, balanced starters. This was followed by those in the second profile, pure starters, while none of the participants from the first profile, mixed starters, mentioned this challenge. This may suggest that teachers with a more intermediate level of autonomous motivation, with or without controlled motivation, may struggle more with adapting to a researcher role while they are busy with their daily teaching routines as vocational teachers. Secondly, a lack of research knowledge and skills was observed as a common challenge for nearly all of the participants, regardless of their motivational profile, making this the most frequent challenge in teacher research. Thirdly, the teacher participants from the first profile, characterised as mixed starters with high levels of autonomous and controlled motivation, tended to express a demand for a supportive research climate, resources, and rewards. Finally, the challenges of workload and family responsibilities were most frequently reported by the participants in the second profile, pure starters. In particular, most of these teachers were females and aged 20 to 40. This may indicate that teachers with high autonomous motivation but low controlled motivation are more likely to view these factors as a significant barrier to increasing their research activities at work.

## 5. Discussion

Our study explored the motivations, challenges, and the corresponding relationships between these factors that vocational teachers face in conducting research, particularly in underdeveloped areas of northwest China. The results suggest that the teachers’ autonomous motivation was consistently higher than controlled motivation, a notable finding given the current policy pressures in China. Furthermore, five distinct categories of challenges were identified: researcher identity, research knowledge and skills, research climate in schools, workload and family care, and resources and financial support. The relationship between motivations and challenges also provided preliminary insights, particularly in how different motivational profiles might influence teachers’ perceptions of challenges in engaging in research activities at work.

### 5.1. Motivations for Teacher Research

Unlike previous studies that utilised a qualitative method to ascertain teachers’ motivations or reasons for conducting research (e.g., [28,39,47], our study applied a person-centred exploratory approach to investigating teachers’ motivation and challenges in carrying out research at work. This research observed unique patterns of Chinese vocational teachers’ autonomous motivation being consistently higher than their controlled motivation in this underprivileged group of participants from Qinghai, indicating that the teachers’ autonomous regulation *might be a key factor* in driving their work on research. The fact that the teachers’ autonomous motivation was higher across all profiles, especially in the second profile, where it significantly exceeded their controlled motivation, is particularly noteworthy in the context of China, where external pressures, such as policy mandates, have played a significant role in shaping teachers’ work behaviour.

Prior studies have suggested that autonomous motivation significantly influences individuals’ positive behaviours, emotions, and beliefs, whereas controlled motivation may lead to adverse outcomes, such as anxiety (e.g., [48,49]). The higher autonomous motivation observed in this study might indicate that vocational teachers in underdeveloped areas, despite facing substantial challenges, are engaging with research positively and are self-driven. This is significant, as it suggests that even though the teachers are under some external pressure, this is not entirely detrimental to their motivation. However, this does not mean that the influence of controlled motivation was too weak to ignore. It appears that a combination of internal interests and external mandates may coexist. Among the three profiles that we identified, almost half of the participants were categorised into the first profile, who showed quite a high level of controlled motivation despite this being relatively lower than their autonomous motivation. This indicates that policy on teacher research, along with autonomy-supportive resources and rewards in China, might strongly impact vocational teachers’ engagement in research at work.

### 5.2. Challenges in Research: Identity, Skills, and Contextual Barriers

Our study captured five key categories in terms of the perceived challenges to research at work for the Chinese vocational teachers. First, a lack of researcher identity was noted as a challenge, especially for vocational teachers in the third motivational profile, balanced starters. These teachers struggled with integrating the researcher role into their professional identity. This is consistent with previous research, which has shown that teachers and school leaders perceive research as disconnected from their primary teaching responsibilities, which might negatively influence their engagement in research [50,51]. Our study also suggests that the researcher identity of the novice and experienced teachers is disengaging. It can be difficult to balance the dual roles of teacher and researcher when facing different work challenges.

Second, a lack of research knowledge and skills was observed as an important challenge across all three profiles, highlighting the need for professional development that specifically targets vocational teachers’ unique needs. Although this finding aligns with those of prior studies [40,52], we made more nuanced findings by categorising specific clusters within this challenge, such as topic selection, theoretical knowledge, research methods, and academic writing. This may suggest that targeted interventions in these areas could significantly improve the research capacity of vocational teachers.

Third, the research climate within schools was found to be a critical barrier, as reported in previous studies [53]. The collectivist culture in China might exacerbate this challenge, where the attitudes and actions of their leaders, colleagues, and other stakeholders significantly influence teachers’ actions. Fourth, work and family care were another challenge that inhibited Chinese teachers from engaging in research, especially for female vocational teachers aged 20 to 40. This finding aligns with previous studies, which have consistently highlighted the heavy workload of Chinese vocational teachers, leading to high levels of job dissatisfaction and turnover intention [54,55]. Heavy teaching loads, coupled with family care obligations, impact more autonomously motivated teachers, who might struggle to balance their autonomous motivation to conduct research with the external pressures of work and family life. One possible approach to addressing these challenges is for institutions to provide more autonomy- supportive work solutions or benefits (i.e., on-site childcare, family educational support, etc.) to sustain vocational teachers’ engagement in research at work.

Finally, a lack of resources and financial support, especially insufficient collaboration between schools and academic universities, was identified as another key barrier. While recent policy initiatives have promoted stronger collaboration between schools and universities [56], our study suggests that such partnerships remain underdeveloped. Teachers expressed frustration with the lack of academic resources and opportunities for collaboration, which appeared to severely impact their ability to engage in meaningful research. This gap underscores the need for institutions to actively promote partnerships and provide vocational teachers with the resources and financial support necessary to maintain their research endeavours.

### 5.3. The Relationship Between Motivation and Challenges

Considering the relationship between motivation and challenges, our results suggested three key trends: the teachers from the mixed starter profile tended to view the research climate and resources and financial supportas major obstacles; research knowledge and skills challenges appeared to be independent of motivation levels; and teachers from the pure starter profile more frequently cited work and family responsibilities as significant barriers. These findings provide preliminary insights into the complex interplay between motivation and perceived challenges in Chinese vocational teachers’ engagement in research at work.

In the profile of mixed starter, where there was strong autonomous and controlled motivation, teachers did not perceive themselves as researchers with great difficulty. However, they were more likely to perceive a lack of institutional support, shown in funding, mentorship, and resources for research, as a very high barrier to the actual undertaking of research. This might be due to high levels of motivation correlating with much higher expectations of the research climate. It is possible that people tend to be much more disillusioned when their external conditions do not align with their levels of motivation [36,57].

The second trend observed was a lack of research knowledge and skills, which was common across all motivational profiles. Regardless of their levels of autonomous or controlled motivation, the teachers reported difficulties with basic research competencies, such as methodology, data analysis, and academic writing. As described by expectancy–value theory, teachers will not complete a task unless they are confident in their ability [58]. This specific finding indeed underscores the importance of targeted professional development since even highly motivated teachers cannot overcome research challenges without the proper training [59,60]. Therefore, improving research skills might be crucial to enable teachers to effectively engage in research. Finally, in the second profile, with high autonomous but low controlled motivation, teachers, especially female teachers aged from 20 to 40 referred to workload and family responsibilities as a hindering factor more often. This suggests a potential conflict between teachers’ willingness to engage in research and the reality of time constrains. This is in line with prior studies, which revealed that workload had a negative impact on teachers’ exploration in other fields (e.g., [61]).

Prior studies have frequently considered motivation and challenges as internal and external factors that can influence teachers’ professional learning and behaviours [62,63], while no studies have ever displayed the possible relationship between these two types of considerations. In our study, we tried to make up for this gap. Based on prior studies, autonomous motivation was often proven to be a positive predictor, while controlled motivation was taken as a negative predictor of teachers’ work and learning beliefs, behaviours, and engagement. Our study, however, provides a different perspective in that autonomous and controlled motivation can be combined in their impact.

## 6. Limitations and Direction for Future Research

The current study has some limitations, which may guide future research. First, the sample in our study was quite small, which may have impacted both the generalizability of the findings and the robustness of the latent profile analysis (LPA). In particular, the participants were all from Qinghai province, which could lead to a regional bias. Future research could benefit from including a larger and more diverse sample to validate these findings further and extend the applicability of the profiles across other underdeveloped provinces.

Second, as this study was conducted in an underdeveloped area, the findings may not necessarily reflect the experiences of teachers from more developed regions. Further research could compare the motivations and challenges faced by teachers in both developed and underdeveloped areas to identify regional differences and provide more targeted recommendations.

Third, this study employed an exploratory approach to investigating the vocational teachers’ motivations and challenges, which means that no definitive conclusions were drawn. The findings provide suggestions and insights for future studies, but the results should be interpreted with caution due to the exploratory nature of the research. Future scholars may expand on these findings by using more comprehensive theoretical and methodological frameworks.

Fourth, since our study used a self-report questionnaire to investigate the vocational teachers’ motivations and challenges in conducting research, there may have been a perception bias. Future scholars could consider other more objective ways, such as on-site observation, or apply more perspectives, such as those of school leaders, to adapt the results.

Lastly, although the results of our study found that a lack of resources and financial support impeded vocational teachers’ engagement in research, we do not know what a comprehensive support system looks like. Therefore, we recommend that future research builds a support system to ensure vocational teachers’ involvement in undertaking research.

## 7. Implications

The exploratory nature of this study provides several implications for both future research and practice. Regarding the former, our study provided three vocational teachers’ motivational profiles for carrying out research, which could be seen as a conceptual model for future research to explore vocational teachers’ motivation for conducting research. In addition, future studies could build on the insights provided by this exploratory study to develop more structured approaches to understanding how various motivational profiles impact teachers’ engagement with research. The linkage between motivation and challenges found in our study may inspire scholars in the future to consider the difference in the challenges for teachers with different motivation profiles. Expanding on these findings, future research could offer more concrete recommendations for promoting research engagement among vocational teachers, particularly in underdeveloped areas.

In terms of their practical implications, since the results suggest that teachers had sufficient autonomous motivation to undertake research, policymakers, school leaders, and other educational practitioners may focus on addressing the challenges that inhibit Chinese teachers from engaging in research.

First, policymakers ought to consider how to develop a comprehensive support system to respond to vocational teachers’ challenges in conducting research. More national, provincial, and municipal policies, such as reducing teachers’ workloads and providing more financial support, could be enacted in order to promote vocational teachers’ engagement in conducting research.

Second, school leaders should recognise the unique challenges vocational teachers face in conducting research, particularly those related to a lack of research knowledge, skills, and resources. Targeted professional development programmes and institutional support could help vocational teachers overcome these barriers, such as helping them develop a researcher identity and increasing their skills in terms of topic selection, theoretical framing, research methods, and academic writing.

Third, this study suggests that female teachers, especially those aged 20 to 40, are more likely to encounter difficulties in balancing research with their work and family responsibilities. Institutions could consider providing more flexible work arrangements, such as on-site childcare and research mentorship programmes, to support these teachers in their research endeavours.

Fourth, the challenge of the research climate in schools may inspire school leaders to promote teacher collaboration in conducting research and seek more cooperation with universities. By doing so, schools could provide professional guidance and establish a reward system to promote engagement in research.

## Figures and Tables

**Table 1:** The results of the fit statistics from latent profile analysis.

Model	AIC	BIC	aBIC	Entropy	aLMR	BLRT
M1	210.22	217.26	204.73			
M2	209.32	221.65	199.72	0.68	0.317	0.217
M3	201.66	219.27	187.94	0.85	0.029	0.000
M4	204.53	227.43	186.70	0.86	0.483	0.600

*Note.* AIC = Akaike information criterion; BIC = Bayesian information criterion; aBIC = adjusted BIC; aLMR = Adjusted Lo–Mendell–Rubin likelihood ratio test; BLRT = bootstrap likelihood ratio test.

**Table 2:** The categories and labels of challenges for participants.

Category	Definition	Number of Participants	Labels
Researcher identity	Teachers’ identity in being a researcher	6	
Research knowledge and skills	Teachers’ perception of their ability to conduct research	28	Topic selection
			Theoretical knowledge
			Research method
			Reporting and writing
Research climate in schools	Teachers’ perception of the climate as promoting them to conduct or hindering them in conducting research	12	
Workload and family care	The amount of effort for teachers to engage in work and family life	11	Teaching workload
			Non-teaching workload
			Family care
Resources and financial support	Support from stakeholders inside or outside schools for vocational teachers	14	Guidance from academic universities
			Extrinsic rewards from schools
			Resources provided by schools

**Table 3:** The frequency of challenges for participants.

Profile	Participant	Researcher Identity	Research Knowledge and Skills	Research Climate in Schools	Workload and Family Care	Resources and Financial Support
1	1-1	0	1	0	0	0
1	1-2	0	0	0	0	1
1	1-3	0	1	1	0	0
1	1-4	0	1	1	0	1
1	1-5	0	0	1	0	1
1	1-6	0	1	0	1	1
1	1-7	0	1	0	0	0
1	1-8	0	1	1	1	1
1	1-9	0	0	1	0	0
1	1-10	0	1	0	0	0
1	1-11	0	1	1	1	0
1	1-12	0	1	1	0	0
Mean		0	0.75	0.58	0.25	0.42
2	2-1	0	1	0	0	0
2	2-2	1	1	0	1	1
2	2-3	0	1	0	0	0
2	2-4	0	1	0	1	1
2	2-5	0	1	1	0	0
2	2-6	0	1	0	1	0
2	2-7	0	1	1	0	0
Mean		0.14	1	0.29	0.43	0.29
3	3-1	0	1	0	1	0
3	3-2	1	1	0	0	1
3	3-3	0	0	1	1	1
3	3-4	0	1	0	0	1
3	3-5	0	1	0	0	0
3	3-6	1	0	1	0	0
3	3-7	0	1	0	1	1
3	3-8	0	1	0	0	0
3	3-9	0	1	0	0	0
3	3-10	0	1	0	0	0
Mean		0.2	0.8	0.2	0.3	0.4

**Table 4:** Descriptive statistics.

Category	Mean	SD	Skewness	Kurtosis
External regulation	2.97	1.25	−0.11	−1.07
Introjected regulation	2.67	1.18	0.59	−0.74
Identified regulation	4.36	0.67	−0.62	−0.96
Internal motivation	3.44	0.94	−0.18	0.16
Autonomous motivation	3.90	0.71	−0.19	−0.58
Controlled motivation	2.82	0.89	−0.25	−0.61

## Data Availability

The data will be made available on request.
